# Prostitutes, sailors and professionals - lived experiences of medical school students and staff with tattoos

**DOI:** 10.12688/mep.19592.1

**Published:** 2023-08-21

**Authors:** Kevin McConville, Shubham Agwan

**Affiliations:** 1 School of Medicine, University of Dundee, Dundee, DD1 9SY, UK

**Keywords:** Tattoos, Professionalism, Medical Education, Policies, Self-Identity, Ethics, Workplace

## Abstract

**Background:** The aim of this study was to explore the lived experiences of medical school students and staff to uncover gaps in policy and its effects on those with tattoos. **Methods:** Adopting a phenomenological design, semi-structured interviews were conducted with ten medical teaching staff and students who had tattoos, within one university medical school. Five key themes emerged: tattoo motives, tattoo content, positive and negative views on tattoos and challenges for establishing policy. **Results:** Findings suggested that no existing stigma towards staff or students exists, however, there is an absence in existing policy regarding tattoos. Participants’ motivations for obtaining tattoos mirrored those of the public, most commonly being artistic expression. Images of tattoos related to nature were common. Views on tattoos suggested the need for a full reform of ‘dress code’ policy for the profession. Several ethically laden scenarios were espoused, highlighting the need for an official stance on tattoos. Policy discussions flagged challenges for those who construct such documents; cultural and generational differences being commonly identified by participants. **Conclusions:** Universally there was uncertainty on current policy, with no individual able to recount specific guidance. The existence of a hidden curriculum surrounding tattoos within medical school further increases the need for guidance reform.

## Introduction

Tattoos have been present for over 5000 years, with a 'Tylorean Iceman' serving as the earliest reported discovery of body markings resembling tattoos ( Jablonski, 2013). Historically, tattoos were restricted to a minority, but are now publicised as elements of one's identity ( Botz-Bornstein, 2015). Tattooing has changed from a practice previously seen in lower socio-economic classes to acceptance in a larger population, including working and professional classes ( Mun *et al.,* 2012).

However, there is still stigma towards tattooed individuals albeit that undergraduate medical students previously familiar with tattoos, through family or friends with body art, display less prejudice towards tattooed strangers than others ( Dickson *et al.*, 2014). Those with negative attitudes towards tattoos are perhaps influenced by assumptions they make or have experienced. Carroll *et al.* (2002) found participants with at least one body modification, such as a tattoo or a body piercing, were substantially more likely to engage in certain taboo behaviours e.g., illicit drug use and sexual behaviour. One consideration regarding negative perceptions of tattooed individuals is the positioning and content of the tattoo ( Resenhoeft *et al.*, 2008), something we note in previous work ( Callaghan & McConville, 2018).

These linkages between prejudice and behaviours are recurring themes. Guéguen (2013) exemplifies this regarding men’s perceptions of tattooed women and sex on a first date. However, people’s preconceived ideas are particularly important when it comes to the topic of tattooed healthcare professionals. Au *et al.* (2013) suggest that participants preferred their doctors to have no visible tattoos upon first meeting. Conversely, Westerfield *et al.* (2012) comment that those receiving healthcare did not view their tattooed healthcare staff any more negatively than those without tattoos. Furthermore, at the time of writing, no current literature exists that explores tattooed professionals’ own reasons or feelings behind acquiring a tattoo.

In considering trust, Au *et al.* (2013) show that the absence of tattoos was a trait identified as important for a good first impression in a minority of participants, yet the presence of this characteristic raised questions of the perception of tattoos in healthcare professionals. Likewise, research with student nurses, colleagues and patients, ranked nurses with visible tattoos to be “the least caring, skilled and knowledgeable” ( Thomas *et al.*, 2010, p. 489). With studies such as this, it is not surprising to assume that most doctors would tend to dress in smart attire, displaying minimal body art when in contact with patients. We assume that these decisions are based upon the risk of damaging perceptions or stigma of the health professional from the public point of view. However, this may only be conjecture, something this research sought to address. Thus, whilst case studies have highlighted the challenges for a professional of self-expression, juxtaposed with cultural sensitivities ( Martin *et al.*, 2019), we question the ethics of whether doctors should be aware of the potential perceptions of their tattoos and try to conceal or cover them, thus risk suppressing their self-identity.

Logically, since one health care professional will see many patients, it might make sense for the doctor to mask their tattoos rather than expecting every patient to have cultural awareness. It is this personal and unique experience with tattoos that this study sought to explore within a phenomenological approach ( van Manen, 2017). As such, the findings aimed to determine if this is an issue suggesting potential for specific policy ruling on how to approach this type of scenario as a professional working in health care.

### Policy and practice

Literature divides policy into ‘policy as text’ and ‘policy as discourse’ and encourages readers to consider policy as a series of processes or outcomes rather than discrete sections ( Ball, 2015). We align with ‘policy as discourse’, i.e., a continuous and flexible process which is often based on several people’s viewpoints whereas ‘policy as text’ is highlighted as the traditional view of policy as strict rules with little to no exceptions.

The General Medical Council (GMC) is the primary governing body outlining healthcare standards and advice, through their policy for medical students, schools, and doctors in the United Kingdom (UK). The National Health Service (NHS) also collaborates with the Department of Health to publish regional policies which can differ regarding local disease epidemiology and treatments ( Scotland, 2020). Local policy development is often impaired by factors such as lack of funding, time and policymakers’ own experiences ( Elliott & Popay, 2000). The latter authors ( Elliott & Popay, 2000) recommend more efficient communication between researchers and local policy developers to provide accurate, evidence-based outcomes in policy. Management of the medical profession is often situated between a struggle in differences in policy derived from members of the profession and members of the state ( Salter, 2007).

When considering medical students in particular, most of their guidance on professionalism comes from either GMC documents or their medical school. Within GMC policy there are several expected standards highlighted. These include *Outcomes for Graduates* ( General Medical Council, 2018), *Good Medical Practice* ( General Medical Council, 2019), *Achieving Good Medical Practice: Guidance for Medical Students ( General Medical Council, 2016a)* and *Professional Behaviour and Fitness to Practise: Guidance for Medical Schools and their Students* ( General Medical Council, 2016b). *Outcomes for Graduates (2018)* emphasises the need to:

*“…recognise the potential impact of their attitudes, values, beliefs, perceptions and personal biases (which may be unconscious) on individuals and groups and identify personal strategies to address this…”*
                                                                                                                ( General Medical Council, 2018, p. 9)

However, the latter fails to explore this in detail with regards to examples of when personal beliefs or attitudes may impact the patient. Likewise, within *Good Medical Practice* ( General Medical Council, 2019) is the concept of making sure the patient is comfortable, but it provides no expansion on associated perceptions of the doctor, instead pursuing a fitness to practice approach. *Good Medical Practice* (2019) does, however, mention that a patient not receiving optimum care due to insufficient policy should be appropriately dealt with by the physician. Whilst this section was perhaps intended with treatments of diseases or conscientious objections in mind, we would argue this raises the ethics of due process e.g., should a patient raise the objective of being uncomfortable due to a visible tattoo, thus highlighting the lack of policy on the matter.

Likewise, *Achieving Good Medical Practice* (2016a) which is aimed specifically at students, reminds a student’s obligation to raise concerns should they notice patient discomfort. Given tattoos are becoming more common, the instances of older generational patients who disapprove of their healthcare providers’ tattoos, could theoretically increase. Thus, reinforcing a need for official governance on tattoos in healthcare settings to avoid patient discomfort. *Professional Behaviour and Fitness to Practise* ( General Medical Council, 2016b) serves to guide medical schools regarding students who have been called into question concerning behaviours, health and well-being, or general professionalism. Whilst this resource provides detail about how to address professionalism concerns at different levels, it does not give aspect on what warrants a low or high-level professionalism concern, thus making it difficult for medical schools to act on, for example, if a medical student was reported for having an offensive tattoo ( General Medical Council, 2016b). Although this is probably not a common occurrence, the concept of policy is underpinned by having protocols in place for all events, including uncommon ones and how to address them. Furthermore, when considering NHS policy, the Scottish Government has a dress code in place for NHS Scotland staff, but this does not mention tattoos ( Government, 2018).

### Aim

This research aimed to contribute a new dimension on the topic of tattooed doctors by building on previous evidence ( Callaghan & McConville, 2018). Specifically, it concentrates on the personal histories of tattooed medical school students and staff to uncover views, understandings, or stigma towards their tattoos through lived experiences.

## Methods

### Study design

This study was qualitative in nature and applied a phenomenological methodology to explore lived experiences of the participants ( Creswell, 2013). We argue that phenomenology was considered as an accurate approach to subjectively identify commonality between the experiences of several individuals ( van Manen, 2017). Therefore, this aligns well with the aims of this research, which lays a path to discover common themes amongst individuals describing their experiences with tattoos.

### Participants and recruitment

Tattooed University of Dundee (UoD) medical school staff or students formed this study’s inclusion criterion making the exclusion criterion, by definition, any member of the public or any UoD medical student or staff without tattoos. Sample size was based on responses to a recruitment email initially sent out in January 2020 to all school staff and students, to maintain an effort to include both cohorts to ensure a fair representation of experiences. Consideration was given to the concept of data power as participants were recruited ( Guest *et al.*, 2006).

Convenience sampling was adopted as the primary recruitment strategy due to the relatively short time scale (January to March 2020) available to carry out data collection in this study ( Robinson, 2014). A ‘gatekeeper’, using UoD medical school professional services staff acted as an intermediary. Two separate emails were sent to all students and staff of the medical school inviting interest. Individuals who responded were provided with a participant information sheet and followed up in line with an approved ethics process.

### Data collection and analysis

Semi-structured, one-to-one interviews were favoured over focus groups as the primary data collection method due the personal and private nature of some participants’ tattoos ( Gill *et al.*, 2008). The use of semi-structured interviews (n=10), all conducted by one researcher (SA), face-to-face on a secure and private campus site (maximum time allowed one hour), allowed flexibility for the researcher to probe and explore a particular participant response, should more depth be required ( Britten, 1995). Questions within the interview guide aimed to encompass topics that the study sought to explore, whilst leaving space for the participant to freely broach other elements ( Turner, 2010). The interview guide (see *Extended data* ( McConville & Agwan, 2023)) was piloted with a small cohort of intercalating Medical Education students. All interviews were audio-recorded and transcribed verbatim ( Bailey, 2008). Participants who consented to photographs of their tattoos being shared e-mail a photograph.being shared e-mail a photograph.

Data analysis was carried out in a reflexive thematic manner ( Braun & Clarke, 2006). Interview transcripts were initially critiqued by one researcher (SA) to identify commonalities, which were then grouped as themes. This was followed by a number of selected reviews by the second researcher (KM). Discussion enabled agreement on the coding and thematic framework. Consideration was given to the concept of member checking ( Birt *et al.*, 2016) but time did not allow for the return of manuscripts to participants for commentary due to the nature of the BMSc Medical Education timelines.

### Ethical considerations

Informed written consent was obtained from participants in accordance with guidelines outlined by the British Educational Research Association (2018). Before the interviews, participants were provided with a participant information sheet, which they had the opportunity to review and agree to via email. Prior to each interview, a written consent form was signed by both the participants and the researcher. This consent form explicitly included consent for the analysis of their transcribed interviews as well as the inclusion of a picture of the participants' tattoo in the study, which might be included in future publications. The study received ethical approval from the School of Medicine Research Ethics Committee SMED Rec 19/165.

## Results

**Figure 1.**
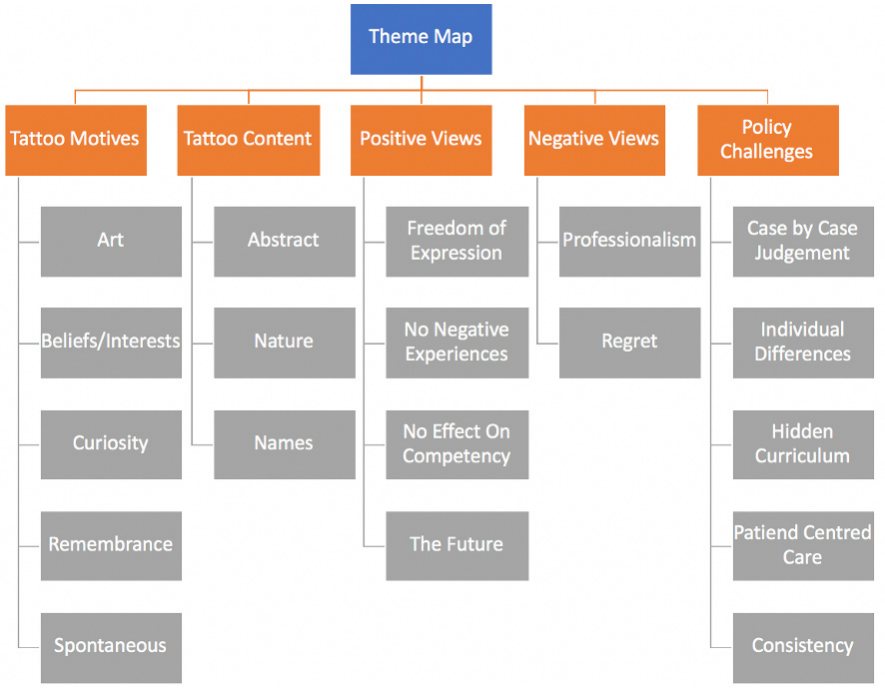
Themes and sub-themes map.

In total, ten interviews were carried out to the point of data saturation ( Guest *et al.*, 2006). Five students and five staff were interviewed to allow a fair representation of each cohort’s views and experiences. Five main themes arose from these, each with multiple sub-themes ( Figure 1).

### Tattoo motives

Motives were often highly personalized and unique. Artwork selected was predominantly valued, thought out and representative of personal beliefs, although there was some occasional spontaneity in image choice.

*“I like them on people, and I like them on me as like a way to be able to express yourself through, you know, art.”*
                                                                                                                                             Student-2
*“I’ve got one which represents my strong feminist beliefs.”*
                                                                                                                                             Staff-5

A common origin of curiosity identified was the idolisation of celebrities or role models who had tattoos themselves. Tattoo use as a remembrance motive was divided into remembering a person or an event.

**Figure 2.**
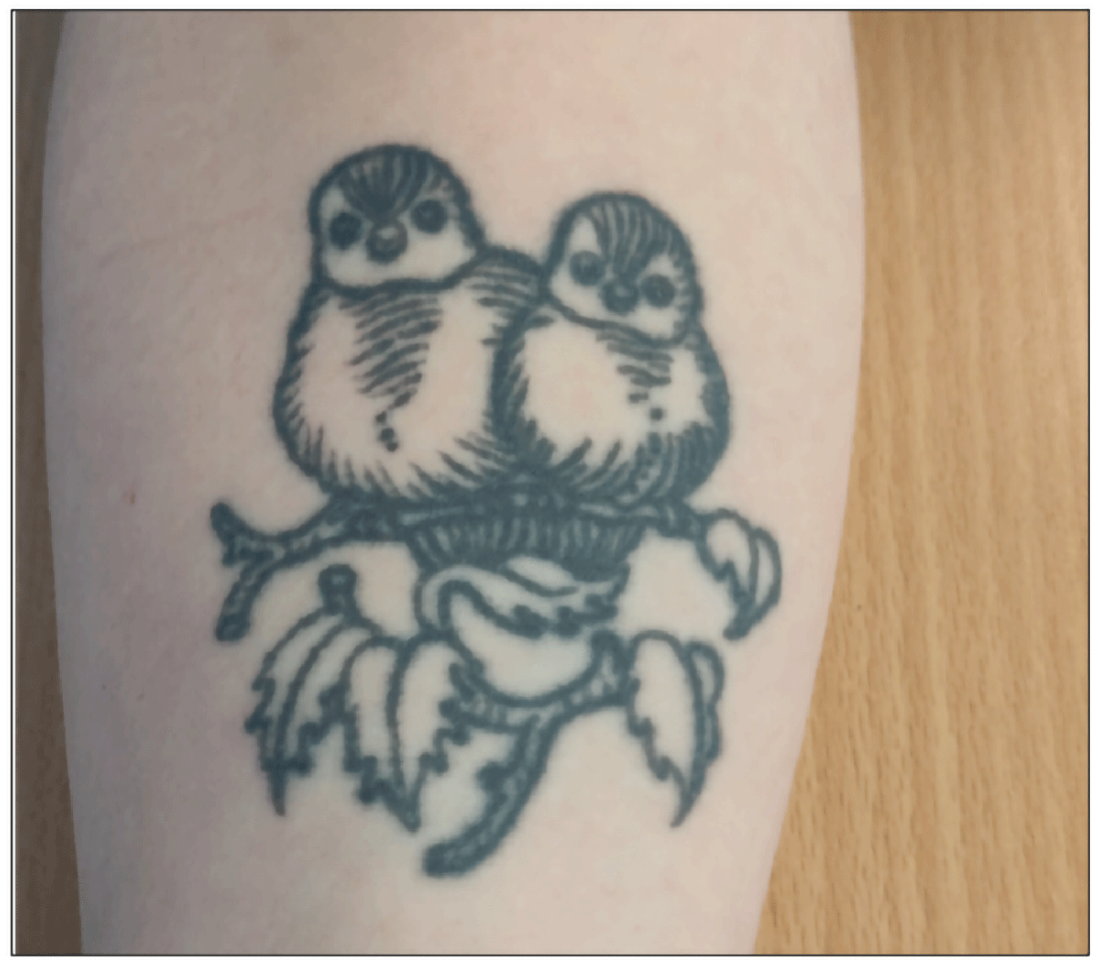
Participant’s hummingbird tattoo on left upper arm.

*“...so, my first tattoo was the hummingbird on my left upper arm* [ Figure 2]. *And I was in America, travelling when I was a teenager when I got it and it was kind of to commemorate that travelling experience.”*
                                                                                                                                             Staff-2

### Tattoo content

Bearing in mind the distinctive and unique nature of tattoos their content seemed to rest within three main categories: abstract, nature and names.

**Figure 3.**
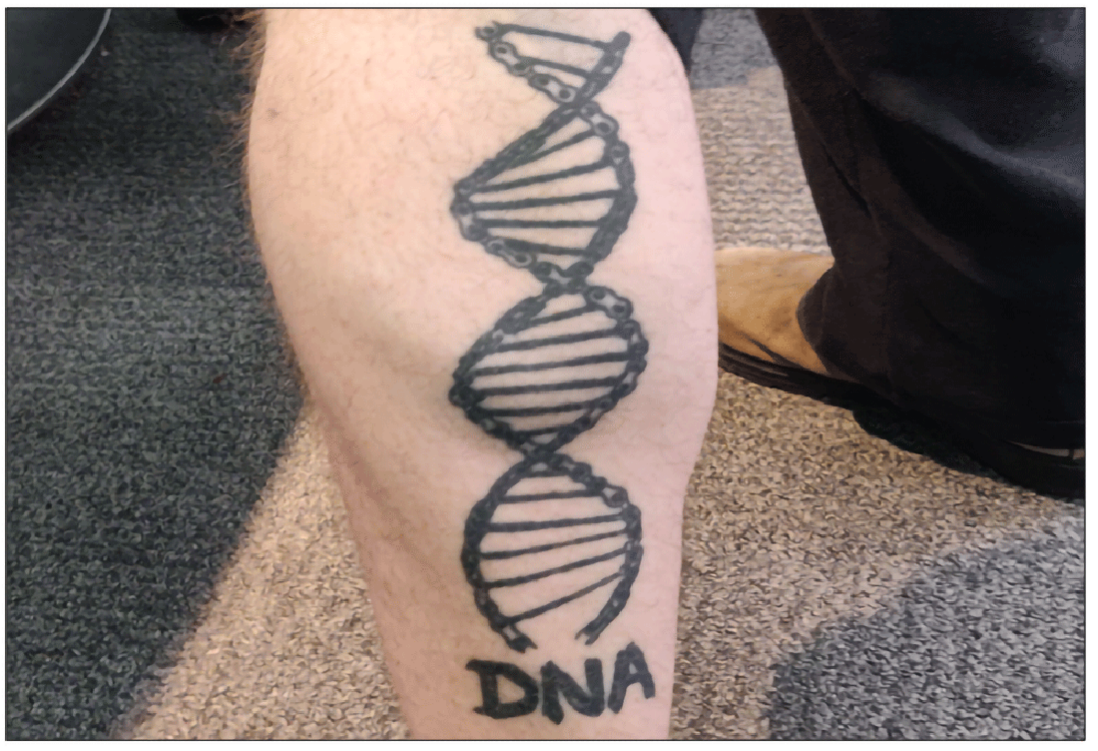
Participant’s DNA double-helix calf tattoo.

*“...as I got older, it was a case of my children's names going on me and my husband's name.”*
                                                                                                                                             Staff-3
*“There's a five-point star with 'I'm a star' and on the foot is a moon and can't remember what it says, 'I'm out of this world' I think...The calf one is a DNA double helix made out of a bike chain.”* [ Figure 3]
                                                                                                                                             Student-5

### Positive and negative views

Overall, there was a positive experience of tattoos. Participants remarked how their tattoos might be used as a route to patient discussions and promoted as a personal signal towards freedom of expression, irrespective of their competency.

*“You can't say to, I don't think you could say to an entire profession that they're not allowed to have tattoos...I think that kind of takes away their freedom”*
                                                                                                                                             Student-1

There were clear indicators that their tattoos were not being perceived in a negative sense, including their levels of competency.

*“I don't think I've been environment where anyone has criticised mine or any other person's tattoos.”*
                                                                                                                                             Student-4

However, reflections on portrayals of professionalism and sometimes regret did emerge at times.

*“…I think if someone made that assumption that because of my tattoos I wasn't able to do my job, I'd be quite offended by that.”*
                                                                                                                                             Staff-2
*“As I've got older, I thought about what I look like on my wedding day with them, I didn't think about that at the time when I got them.”*
                                                                                                                                             Staff-3

### Policy changes

A need for policy was drawn to attention by the uncertainty of the participants on existing guidance. Views deliberated the utility in a universal policy, versus judgements made on a case-by-case basis.

*“I think it would be impossible to write a list of what is offensive because offence is subjective, it's personal … it should be a case by case thing and if it comes to be an issue it should be explained then.”*
                                                                                                                                             Staff-5

Cultural and generational differences held sway, underpinned by influences from within a hidden curriculum.

*“...my gran to myself even says, 'well, it's only like prostitutes and sailors who have tattoos' because in her day that's what it was. Whereas now it's much more accepted that everyone and anyone has tattoos really...”*
                                                                                                                                             Student-1

## Discussion

The lived experiences of medical school staff and students with tattoos unearths a complex mix of themes and sub-themes. In our research the topic of tattoos among medical school staff and students has sparked intriguing reflections, revealing a rich tapestry of lived experiences. This exploration has unveiled a multitude of themes and sub-themes that shed light on the intricate dynamics surrounding tattoos within the medical community. From the motives behind acquiring tattoos to the content chosen, and from the contrasting perspectives of positivity and negativity toward tattoos, to the implications of institutional policies, this multifaceted inquiry unearths a complex interplay of attitudes and beliefs. By delving into the intricacies of these themes, we can gain a deeper understanding of how tattoos intersect with the lives and identities of medical professionals and learners, and their implications for the broader medical education landscape.

### Tattoo motives

Fewer students than staff identified art as a specific motive, which was an unexpected finding, despite younger generations having a theoretically greater interest ( Scaglione, 2018). However, ‘art’ as a common reason aligns well with prior evidence ( Wohlrab *et al.*, 2007). The same was true of ‘individuality’ as a purpose for tattoos. Our participants had tattoos representing individual and religious beliefs, a factor which also mirrors Martin, Cunningham and Agyemang-Dua (2019) whom outlined miscommunications relating to a religious tattoo. However, this was not a problem that has been reported to date within our cohort. Akin to beliefs and interests, our research noted a general curiosity in tattoos and body art as a motive not dissimilar to Forbes (2001) who found ‘just liked the look of it’ and ‘to be like a friend’ to be a common viewpoint.

Perhaps maturity lends itself to life experience and therefore remembrance, which was a motive identified by a greater proportion of staff compared to students. This may simply be down to the staff being older in general and thus having more life events to commemorate via body art. Comparing this to Forbes’ (2001) findings on student tattoo motives, we see in our participants that ‘remembering a life event’ occurred less (n=4). In contrast, Wohlrab, Stahl and Kappeler (2007) note that ‘personal narrative’ exists as a predominant theme. Oksanen and Turtiainen (2005) argue that significant life changes within people’s histories are often made easier to come to terms with for those who document them upon their body. Records of art that were a spontaneous decision with no significant prior thought were identified as a motivation within our work (n=6). Looking back on Wohlrab, Stahl and Kappeler (2007) we note that ‘no particular reason’ was the closest theme in keeping with our research.

### Tattoo content

Participants’ abstract tattoos ranged from a phrase with a small emblem to a sailboat. Like Resenhoeft, Villa and Wiseman (2008) these examples can be considered to be inoffensive and not intimidating and thus are less likely to provoke any negative response from others. Naturistic type tattoos mostly consisted of flowers or small animals such as butterflies, again thus considered relatively unoffending. Whilst no literature could be found on the prevalence of ‘nature’ content tattoos, indicators of tattoos in the general population suggest it might well be commonplace ( Kluger *et al.*, 2019).

Similarly, ‘people’s names’ are a content category not identified in literature and from our research seems an uncommon thing to have tattooed, at least within the medical staff and student cohorts. Whilst none of the tattoos within this study were considered ‘offensive’ by the primary researcher, this does call into question the ethics of protocols in place for such tattoos. Given that personal opinions should be respected in an expert workplace, if a colleague or patient was to take offence to one of these tattoos, policy does not outline an approach in sufficient detail.

### Positive and negative views

Akin to communication, tattoos as a method of ‘freedom of expression’ were identified by nine of the ten participants and reinforced strongly by Forbes (2001). Our findings also align with Vail (2000), who record that tattoos have become one of the primary methods of self-expression in modern society. The GMC suggests within its *Good Medical Practice* ethical guidance:

*“You must not express your personal beliefs (including political, religious and moral beliefs) to patients in ways that exploit their vulnerability or are likely to cause them distress.”*
                                                                                                                ( General Medical Council, 2019, p.18)

This quote questions where a line can be drawn between when a patient is distressed and when a physician is not allowed their right to freedom of expression via their body art; something that was previously argued by Callaghan and McConville (2018). Regarding tattoos as a method of communication, one student was able to describe a specific experience involving an autistic child who would only interact with the students. Historically, tattoos have been used to convey negative messages such as the ‘D’ tattooed on military deserters ( Brouwer, 1998). Doss and Ebesu Hubbard (2009) found that a tattoo with greater communicative value is often noticed more readily by others.

Our work did not elicit any negative experiences nor commentary regarding low competency attainment. This suggests agreement with Westerfield *et al.* (2012) who found that most patients did not identify the absence of tattoos on their care provider as something they sought out. However, we note that Westerfield *et al*.’s (2012) findings focused on nurses with tattoos rather than doctors in training. This discrepancy between healthcare professional expectations was alluded to by one staff participant, who said they wouldn’t notice a tattooed nurse in particular but would with a tattooed doctor, perhaps highlighting an inconsistency in approaches underpinned within policy or hidden curricular. Our findings thus conflict with Madera and Hebl (2012) who show that visible stigmas can lead to discrimination within job application rankings. However they highlight, that while it has been established that nurses rank tattooed colleagues to be significantly less competent ( Thomas *et al.*, 2010), perceptions of different tattooed professions thus exist.

All participants had a positive outlook on the future. One staff member predicted that one day, it would be more common for someone to have a tattoo than not. This trend would be in line with the ‘tattoo renaissance’ described by Mun, Janigo and Johnson (2012). One staff member mentioned that the acceptance of tattoos came from more role models donning them in modern society, MacCormack (2006) reports the same.

Regarding negative views on tattoos and professionalism, seven participants said that whilst tattoos shouldn’t be seen as unprofessional, this may be the case on occasion. This viewpoint can be related to Baumann, Timming and Gollan (2016) who found that tattooed surgeons were perceived more negatively than the same tattooed individual as a mechanic. This again illuminates the higher expectations of the public when it comes to doctors, compared to other professions. The concept of professionalism was also a key finding within earlier research ( Callaghan & McConville, 2018) that highlighted this missing topic from GMC policy regarding tattoos. The GMC state:

*“You must make sure that your conduct justifies your patients’ trust in you and their trust in the profession.”*
                                                                                                                ( General Medical Council, 2019, p. 21)

Upon closer examination of the dress code policies of the 14 health boards across Scotland, the word ‘tattoo’ is only found in half of these policy documents ( Borders, 2019; Galloway, 2016; Lanarkshire, 2015; Lothian, 2015; Valley, 2016). These five health board policies make clear that tattoos which are deemed offensive should be covered up, with NHS Shetland (2018) deeming a tattoo to be dealt with as an open wound to be risk assessed on how to cover up. NHS Lanarkshire (2015) pushes for visible tattoos to be discouraged and if present, to be covered up if found to be offensive. NHS Borders (2019) also states that any tattoo deemed offensive should be covered up, but interestingly exemplifies offensive tattoos as ‘religious, sexual or football-related’, and is the only one of the fourteen health boards to do so. This draws into question whether these examples are open to interpretation, especially since some religious, sexual or football beliefs could be seen to be highly personal and emotional by some. NHS Forth Valley (2016) are the only health board which take religious tattoos into particular consideration, saying that certain religious henna tattoos can be deemed appropriate, in accordance with the department lead. This highlights a scope for potential fitness to practice cases, simply due to a lack of policy. The extremely notable finding within our study of six participants using the swastika as an example of an unacceptable tattoo fits entirely with the premise reported by a case study which outlined a similar issue ( Martin *et al.*, 2019).

A point made by one student was that she did not regret the tattoo itself but rather that it did not turn out the way they had envisioned it. This finding matches with Sanders (1985) who argued that most individuals with tattoo regret credit this to dissatisfaction with the execution, rather than the tattoo itself. This same student also went on to disclose that she was firstly unable to tell her parents but grew to like her tattoo more. This disagrees with Swami’s (2011) findings, which state that women tend to have increased appearance anxiety three weeks post-tattoo.

### Policy challenges

All our participants expressed uncertainty towards knowledge of existing policy concerning tattoos within the medical field. GMC documents for medical schools and students reiterate the importance of the comfort of patients and the role of the doctor to uphold professional standards, however, none of these documents explore this with regards to tattoos or even a dress code ( General Medical Council, 2018; General Medical Council, 2019; General Medical Council, 2016a; General Medical Council, 2016b). The general acceptance of tattoos is a topic which one would expect to find within local university guidelines, however upon further exploration, the University of Dundee Medical School policies do not address tattoos.

Close examination of local Scottish NHS health boards reveals a recurring absence of tattoo policy as highlighted above. Salter recommended the only way to provide healthcare professionals with a complete policy addressing tattoos was to develop and improve communication between policymakers within the professional governing body and policymakers within the state governing body ( Salter, 2007). The longer that gaps in policy remain, the greater is the risk of unnecessary cases of fitness of practice arising. Whilst there are no documented cases of tattoo-related unprofessionalism claims escalating to tribunal within the medical profession, there are some in other professions which highlight the potential for problems to arise ( Swami, 2011).

One major challenge facing policymakers is the concept of every tattoo complaint being considered on a case-by-case basis. This is because tattoos are often individualistic and hold specific meaning to the beholder; one which may not mean the same to another person ( Martin *et al.,* 2019). Would a tattoo based on an individual’s protected characteristics e.g., religious beliefs, collide with patient care? This makes it almost impossible to create comprehensive ‘acceptable’ and ‘not acceptable’ tattoo lists and requires thought to a fair approach. Similarly, a strongly expressed opinion was in the difference in generations with regards to tattoo perceptions. All participants said that they felt older patients generally had a more negative outlook on tattoos compared to younger patients ( Mun *et al.,* 2012). As such, this is an additional challenge for policymakers to consider. Is it possible to construct governance which caters to the opinions of all generations or is some sort of ‘middle-ground’ compromise required?

The majority of our participants alluded to ‘a feeling’ they perceived about certain tattoos or expressed a level of ‘common sense’ amongst medical students when obtaining tattoos. An example was a student saying that they felt that face and neck tattoos were unprofessional but were unable to explain why. This may be explained by the concept of the hidden curriculum ( Hafferty & Franks, 1994). With no official policy on tattoos, these assumptions made by the participants highlight the presence of a hidden curriculum, which can often be dangerous and lead to incorrect decision making. Finally, when it comes to policy, it is crucial to emphasize the importance of considering the long-term implications of tattoos for undergraduate students. We propose that policies should promote consistency throughout a medical career and among various members of the extended multi-disciplinary team. By addressing the issue of tattoos during undergraduate education, students can make informed choices that do not conflict with their future postgraduate clinical work. Ensuring alignment between undergraduate guidelines and the expectations of postgraduate practice is essential for a seamless transition and successful professional development.

This study acknowledges several potential sources of bias and limitations that may influence the findings. One area of concern is the voluntary nature of participation, which suggests that the individuals who volunteered for interviews may possess stronger opinions on tattoos compared to the general population of medical students and staff. Moreover, the personal views of the primary researcher (SA) on tattooed doctors could have influenced the data analysis and thematic emergence. To mitigate these biases, reflexivity was employed, allowing the researcher to balance intervention during interviews while representing participants' views without imposing personal bias. It is important to note that the primary researcher did not have any tattoos during the study whilst KM does. The use of semi-structured interviews helped minimize bias by ensuring consistent exploration of key questions across cases while still allowing for diverse personal and professional discussions. However, a limitation of the study was the inability to revisit participants for additional data collection, as is typically done in phenomenology. This constraint, driven by time limitations, should be considered when interpreting the study's findings.

## Conclusion

This study adopted a phenomenological approach to explore the lived experiences of tattooed medical school staff and students. Overall, our findings indicate that medical school staff and students at the University of Dundee are not discriminated against or judged for their tattoos. In terms of their tattoos, they have similar motivations to that of the general public ( Wohlrab *et al.*, 2007). The content of their tattoos typically also aligns well with views held by the wider population ( Resenhoeft *et al.,* 2008). However there appears a disparity between professional roles and perceptions e.g., doctors vs. nurses. A lack of policy within the university or the NHS risks ethical and legal challenges in the future.

## Data Availability

The raw transcripts from interviews cannot be shared as participants consented only to their pictures and/or selective illustrative quotes being used. Discovery: Interview Guide used in Prostitutes, sailors and professionals - Lived experiences of medical school students and staff with tattoos https://doi.org/10.15132/10000234 ( McConville & Agwan, 2023) This project contains the following extended data: Interview_guide.docx Data are available under the terms of the Creative Commons Attribution 4.0 International license (CC-BY 4.0).
