# Supplementary material for: Prostitutes, sailors and professionals - lived experiences of medical school students and staff with tattoos
Source: MedEdPublish (2016). 2023 Aug 21;13:42. [Version 1] doi: 10.12688/mep.19592.1 (PMC10835103; doi:10.12688/mep.19592.1)
Supplement: Peer review history

# Reviewer report: Reviewer response for version 1

**DOI:** 10.21956/mep.20989.r35819

**Published:** 2024-02-15

**Authors:** Charlotte Dann

**Affiliations:** 1 Department of Psychology, University of Northampton, Northampton, England, UK

Thank you for an interesting paper, and focus on a much needed area (the presentation of 'professional' in healthcare settings does need more interrogation).

 I would be wary of the presentation of 'cause and effect' type research, such as Carroll et al (2002), as a correlation between tattoos and risk taking does not equal causation (given they were surveying for risk taking behaviours and tattoos, the link was already there through what they were looking for). I am conscious of the overly negative portrayal of tattooed bodies via quantitative methods that needs more critical thought when it comes to presentation.

 Some odd selection of argument in relation to healthcare (why include dated research on tattoos, sex, and dating? The relevance isn't clear).

 I am confused by the inclusion of a phenomenological approach, but then using RTA as a method (i.e. rather than IPA?) could the connections between phenomenology and RTA be explained here.

 It would be good to see more participant detail here, beyong medical student/staff identity. Research by Dann (2021) and Thompson, detail the gendered nuances in particular with tattoos, but also issues such as age and class.

 The themes, as presented, could be developed further. From what I see presented, for example- the quotes from the 'positive and negative' views speaks more to issues of agency, ability, and social norms, than to just positive and negative views.

 The challenges to policy aspect is interesting - it would be beneficial to see the tattoo interview analysis and the comments regarding policy better combined, talking to each other - at the moment, they're presented as separate issues.

Have any limitations of the research been acknowledged?

Yes

Is the work clearly and accurately presented and does it cite the current literature?

Partly

If applicable, is the statistical analysis and its interpretation appropriate?

Not applicable

Are all the source data underlying the results available to ensure full reproducibility?

Partly

Is the study design appropriate and is the work technically sound?

Partly

Are the conclusions drawn adequately supported by the results?

Partly

Are sufficient details of methods and analysis provided to allow replication by others?

Partly

Reviewer Expertise:

Tattooed bodies, femininities, social class, identity, gender, qualitative methods, critical psychology

I confirm that I have read this submission and believe that I have an appropriate level of expertise to confirm that it is of an acceptable scientific standard, however I have significant reservations, as outlined above.

# Reviewer report: Reviewer response for version 1

**DOI:** 10.21956/mep.20989.r35818

**Published:** 2024-02-01

**Authors:** Manda Sexton

**Affiliations:** 1 Kennesaw State University, Kennesaw, Georgia, USA

I consider this to be a well-executed study and write-up of results. The language is clear and lacks jargon that would be a barrier to those outside the medical fields, such as myself. This article also raises some great questions and concerns for policy makers in the medical fields as body modifications are becoming more and more common.

 There were a few concerns I have with the study. First, I do wish there had been member checking applied as it is best practice in this type of study to promote dependability and credibility in qualitative studies. The "time constraint" is mentioned multiple times, however as someone outside of this field, I am still unsure *why* there is a time constraint that would impair the very important credibility of the study results. This is especially true as there seems to be one researcher doing the majority of the analysis with another verifying, which will lend itself to follow any bias the researcher might have on the subject coming into the study. However, both of these concerns were addressed in the bias section appropriately. Perhaps just a tiny bit more detail on why there was a time constraint would benefit this paper.

 Another concern is the applicability of the study results outside the western world. This paper is very focused on a particular culture and medical/university system found in the UK. If this study were to be repeated elsewhere, the results might be very different. It would be in the researcher's best interest to note early in this paper that they are focusing on only the UK systems and culture. In that vein, I would have liked to see a few actual statistics of the increase in tattoos in the UK (and Scotland would be even better), which is alluded to throughout the paper. 

 Lastly, this paper could use someone to go through it for basic copy-editing. There are a few sentences that are redundant and some word issues. The literature review reads a bit choppy with sentences hanging on their own. But those do not detract from the paper, it is merely a suggestion.

 All around, I found this to be a fine study that begs interesting and timely questions regarding tattoos in medical students and staff. Well done.

Have any limitations of the research been acknowledged?

Yes

Is the work clearly and accurately presented and does it cite the current literature?

Yes

If applicable, is the statistical analysis and its interpretation appropriate?

Not applicable

Are all the source data underlying the results available to ensure full reproducibility?

No source data required

Is the study design appropriate and is the work technically sound?

Yes

Are the conclusions drawn adequately supported by the results?

Yes

Are sufficient details of methods and analysis provided to allow replication by others?

Yes

Reviewer Expertise:

I am a qualitative methodologist that studies body modifications, alternative dress, and professionalism in customer service fields in the USA.

I confirm that I have read this submission and believe that I have an appropriate level of expertise to confirm that it is of an acceptable scientific standard.

# Reviewer report: Reviewer response for version 1

**DOI:** 10.21956/mep.20989.r34591

**Published:** 2023-10-12

**Authors:** Bridgette Masters-Awatere

**Affiliations:** 1 School of Psychology, The University of Waikato, Hamilton, Waikato, New Zealand

There are some typographical errors noted in the article that would be beneficial to remove. Beyond description of staff or student there is minimal description such as gender, ethnicity or age that may have helped contextualise for the reader the perspective of participants in this study. The point about ethnicity becomes additionally relevant as there was no cultural consideration or exploration amongst the participants. Furthermore, there was minimal acknowledgement, or explanation, regarding the lack of ability to allow participants to review their own transcripts or notes.

Have any limitations of the research been acknowledged?

Yes

Is the work clearly and accurately presented and does it cite the current literature?

Yes

If applicable, is the statistical analysis and its interpretation appropriate?

Not applicable

Are all the source data underlying the results available to ensure full reproducibility?

No source data required

Is the study design appropriate and is the work technically sound?

Yes

Are the conclusions drawn adequately supported by the results?

Yes

Are sufficient details of methods and analysis provided to allow replication by others?

Yes

Reviewer Expertise:

Health, Indigenous psychology, patient and clinician experiences of hospital care

I confirm that I have read this submission and believe that I have an appropriate level of expertise to confirm that it is of an acceptable scientific standard.
